# Urgent ureterorenoscopy as a primary treatment for ureteral stone: why not?

**DOI:** 10.1007/s00240-024-01569-0

**Published:** 2024-04-23

**Authors:** Ramazan Uğur, İlyas Yağmur

**Affiliations:** 1 Department of Urology, University of Health Sciences, Başakşehir Çam and Sakura City Hospital, Başakşehir Olympic Boulevard Road, 34480 Başakşehir, Istanbul, Turkey; 2 Department of Urology, Yenişehir, Viranşehir State Hospital, Viranşehir – Ceylanpınar Street, No:3, 63700 Viranşehir, Şanlıurfa, Turkey

**Keywords:** Ureteral stone, Primary treatment, Ureteroscopy, Urgent

## Abstract

To evaluate the feasibility of urgent ureteroscopy (uURS) and elective ureteroscopy (eURS) in the management of patients with renal colic due to ureteral stones. Patients who were operated for ureteral stones between September 2020 and March 2022 were determined retrospectively. The patients who were operated within the first 24 h constituted the uURS group, while the patients who were operated after 24 h were classified as eURS. No limiting factors such as age, gender and concomitant disease were determined as inclusion criteria. Patients with bilateral or multiple ureteral stones, bleeding diathesis, patients requiring emergency nephrostomy or decompression with ureteral JJ stent, and pregnant women were not included. The two groups were compared in terms of stone-free rate, complications, and overall outcomes. According to the inclusion–exclusion criteria, a total of 572 patients were identified, including 142 female and 430 male patients. There were 219 patients in the first group, the uURS arm, and 353 patients in the eURS arm. The mean stone size was 8.1 ± 2.6. The stone-free rate was found to be 87.8% (502) in general, and 92 and 85% for uURS and eURS, respectively. No major intraoperative or postoperative complications were observed in any of the patients. Urgent URS can be performed effectively and safely as the primary treatment in patients with renal colic due to ureteral stones. In this way, the primary treatment of the patient is carried out, as well as the increased workload, additional examination, treatment and related morbidities are prevented.

## Introduction

Ureteral stone is at the forefront of urological emergencies. Classically, it manifests itself as a flank pain phenomenon with acute onset, sometimes spreading to the groin area, accompanied by nausea and vomiting [1, 2]. Fever is not usually seen in the early period unless there is inflammation. It may accompany hematuria and may even be the first symptom in some patients. Pain may also vary depending on the level of the stone in the ureter. For example, while stones in the upper and middle ureter classically give symptoms as mentioned above, distal ureter and especially ureterovesical junction stones present with cystitis-like symptoms such as dysuria, urgency, and frequensy. Absence of peritoneal irritation findings is important in terms of excluding other related pathologies [3].

In the patient who comes with the above-mentioned symptoms, the costovertebral angle sensitivity is checked first by examining the patient, vital signs are evaluated, kidney function tests, acute phase reactants and urinalysis are requested. In imaging, although the sensitivity and specificity of ultrasonography (US) has been shown to be lower than non-contrast-enhanced computed tomography (NCCT), initial US is useful for diagnosing obstruction and planning subsequent diagnostic and therapeutic actions. The definitive diagnosis is made by NCCT and allows us to objectively evaluate the size, localization, number and density of the stone. In addition, renal and ureteric anatomy, hydroureteronephrosis, and pyelonephritis are evaluated with NCCT [4–6].

After the diagnosis of ureteral stones, analgesia of the patient is provided first. What needs to be decided later is which treatment will be given to which patient. Classical treatment of acute renal colic in clinically stable patients without suspected sepsis, according to stone-related factors; medical expulsive therapy (MET), extra corporeal shock wave lithotripsy (ESWL) or ureteroscopy (URS). Percutaneous nephrostomy (PN) or ureteral JJ stent should be inserted in patients with obstructed stones whose pain cannot be controlled with analgesics and clinically suspected sepsis. Then, elective treatment should be performed [7]. Ideally, in ureteral stone management, complete stone clearance is achived as quickly as possible with minimal morbidity.

In this study, we aimed to compare urgent URS (uURS) and elective URS (eURS) in terms of stone-free (SF), complications and overall results as primary treatment for ureteral stones.

## Materials and methods

Ethical approval was obtained from our institution for study. Then, patients who were treated for ureteral stones between September 2020 and March 2022 were evaluated retrospectively through the hospital online database and patient files. Patients who were operated within the first 24 h were classified as uURS, and those operated after 24 h (24 h–21 days) were classified as the eURS group. All patients who had only one stone in the ureter on one side and did not develop secondary to previous ESWL, URS or percutaneous nephrolithotomy were included in the study. No limiting factors such as age, gender and comorbidities were not determined as inclusion criteria. Patients with bilateral or multiple ureteral stones, bleeding diathesis, presence of obstructive pyelonephritis and associated sepsis requiring urgent percutaneous nephrostomy/double JJ stent decompression and pregnant women were not included. While urinalysis is performed in the vast majority of patients (503–87.9%), urine cultures were obtained from patients with acute phase elevation and clinically likely sepsis. Sepsis criteria were defined as the presence of systemic inflammatory response syndrome and confirmed or suspected infection. General clinical status and renal functions were evaluated by looking at complete blood count, urea, creatinine, electrolytes and other biochemical parameters. All patients received antibiotic prophylaxis with group 2 or group 3 cephalosporins. Parenteral forms of these antibiotics were preferred for rapid effect.

Definitive diagnosis and surgery decision were made according to NCCT. The stones were divided into four groups as proximal, middle, distal and ureterovesical junction (UVJ) stones. All surgeries were performed under general anesthesia. The surgeries were performed with 4.5/6.5 and 6/7.5 Fr Richard Wolf and 6.5/7 Fr Karl Storz semirigid ureterorenoscops. We preferred saline solution (NaCl 0.9%) as the type of irrigation. Pneumatic and laser lithotripters were used for lithotripsy. We used a 35 W Holmium:YAG laser as a laser lithotripter. Low energy 0.2–1 J, and high frequency 15–30 Hz were frequently used to avoid push-back of the stone, to avoid perforation in the ureteral wall, and to prevent fiber damage. A basket catheter was also used in suitable small fragments. Ureteral JJ stent placement was decided according to the stone and operative variables during the operation. Early SF and JJ stent status were evaluated in the direct urinary system radiograph taken on the first day after the operation. Stone-free status was considered as the absence of any stone opacity in the NCCT taken at post-operative 4th week.

### Statistical analysis

Mean, standard deviation, median, minimum, maximum value frequency and percentage were used for descriptive statistics. The distribution of variables was checked with kolmogorov-simirnov test. Mann–whitney *U* test was used for the comparison of quantitative data. Chi-Square test was used for the comparison of qualitative data. SPSS 28.0 was used for statistical analyses.

## Results

Of all patients, 75.2% (430) were male and 24.8% (142) were female. While there were 219 patients in the Urgent URS group, there were 353 patients in the eURS group. The mean overall age was 37.85 ± 12.9, 38.1 ± 13.6 in uURS, 37.7 ± 12.5 in eURS (*p* = 0.833). There was no statistically significant difference between the groups in terms of gender, BMI, side of the stone, stent use, serum creatinine, serum reactive protein (CRP) and complications (*p* > 0.05). The hospital stay was 1.51 ± 0.6 days in all patients (Tables 1, 4, and 5).

**Table 1:** Patients characteristic

	Min–Max	Median	Mean ± sd/*n* (%)
Age	6.00–85.00	36.00	37.85 ± 12.92
Gender			
Female			142 (24.8)
Male			430 (75.2)
Body mass index	15.70–37.00	25.80	25.79 ± 3.11
Stone size (mm)	5.00–20.00	7.00	8.06 ± 2.62
Serum creatinine (mg/dL)	0.50–4.1	0.90	1.18 ± 4.24
White blood cell (10^3^/µL)	3.36–25.00	9.00	9.43 ± 2.98
C-reactive protein (mg/L)	2.00–234.00	2.00	7.70 ± 21.99
Operative Time (min)	25.00–75.00	40.00	39.72 ± 9.69
Length of hospital stay	1.00–6.00	1.00	1.51 ± 0.64

The mean stone size was 8.06 ± 2.62 (5–20 mm) overall, while it was statistically significantly larger in the eURS (7.46 ± 2.20 in the uURS and 8.44 ± 2.79 (*p* < 0.000) in the eURS) (Tables 1 and 5). When we look at the stone localizations, more than half of the stones (299 patients, 52.3%) were found to be distal ureteral stones and the number of patients was 94,112,299,67 as proximal, middle, distal and UVJ stones, respectively (Table 2). When we look at the distribution between the groups, the rate of proximal ureteral stones in eURS and distal ureteral stones in uURS were statistically significantly higher (*p* < 0.05), while the ratio of mid-ureter and UVJ stones was similar (*p* > 0.05) (Table 5). The total SF rate was 87.8% (502/572), 92.2% in uURS, and 85.0% in eURS, and a statistically significant difference was observed between the groups (*p* < 0.010) (Tables 3 and 5). The reason for the difference in the SF rate was thought to be the larger stone size in eURS and the excess proximal ureteral stone rate, which has a relatively low probability of SF compared to other localizations.

**Table 2:** Stone parameters and laboratory variables

	*n* (%)
Stone location	
Proximal	94 (16.4)
Middle	112 (19.6)
Distal	299 (52.3)
Ureterovesical junction	67 (11.7)
Side of stone	
Right	268 (46.9)
Left	304 (53.1)
Urine test	
Yes	503 (87.9)
No	69 (12.1)
Urinary nitrite	
(−)	496 (99)
(+)	7 (1)
Urine culture	
Yes	335 (58.6)
No	237 (41.4)
Urine culture growth	
Yes	5 (1)
No	330 (99)

**Table 3:** Operative and postoperative determinations

	*n* (%)	
Stone free		
Yes	502 (87.0)	
No	70 (12.2)	
Failure of access		
No	549 (96.0)	
Yes	23 (4.0)	
Reason for failed access		
Tight ureter	14 (61)	
Mucosal oedema	3 (13)	
Haematuria	1 (4)	
Impacted stone	5 (22)	
Complications		
No	545 (95.3)	
Yes		
Hematuria > 24 h	3 (11)	27 (4.7)
Fever	4 (15)	
Stone migration	14 (52)	
Intensive care unit	2 (7)	
Renal colic	4 (15)	

There was a statistically significant difference between the groups in terms of failure of access, operative time, length of hospital stay and white blood cell (WBC) (*p* < 0.05) (Tables 4 and 5). While failed access was detected in 23 patients in total, tight ureter was the most common cause (Table 3). The reason for the failure of access, operative time and length of hospital stay to be high in eURS was thought to be related to the high stone size and proximal stone ratio in eURS. On the other hand, it was thought that the difference in WBC was caused by the use of non-steroidal anti-inflammatory drugs whose mechanism of action is based on prostaglandin synthesis inhibition. Inhibiting prostaglandin synthesis shows its effect by reducing renal blood flow, diuresis, ureteral smooth muscle activity and local ureteral inflammation.

**Table 4:** Comparison of patient characteristics and laboratory parameters of the two groups

	uURS		eURS		*p*	
	Mean ± sd/*n* (%)	Median	Mean ± sd/*n* (%)	Median		
Age	38.1 ± 13.6	35.0	37.7 ± 12.5	36.0	0.833	m
Gender						
Female	57 (26.0%)		85 (24.1%)		0.600	X^2^
Male	162 (74.0%)		268 (75.9%)			
Body mass index	25.82 ± 3.31	25.90	25.77 ± 2.99	25.80	0.989	m
White blood cell (10^3^/uL)	10.04 ± 3.51	9.40	9.05 ± 2.53	8.70	***0.001***	m
C-reactive protein (mg/L)	11.08 ± 29.79	2.00	5.60 ± 14.95	2.00	0.241	m
Serum creatinine (mg/dL)	1.07 ± 0.49	0.90	1.24 ± 5.38	0.90	0.060	m
Urine culture						
No	142 (64.8%)		95 (26.9%)		***0.000***	X^2^
Yes	77 (35.2%)		258 (73.1%)			
Urine culture growth						
No	76 (98.8%)		254 (98.5%)		0.467	X^2^
Yes	1 (1.2%)		4 (1.5%)			
Urine test						
No	4 (1.8%)		65 (18.4%)		***0.000***	X^2^
Yes	215 (98.2%)		288 (81.6%)			
Urinary nitrite						
(−)	214 (99.5%)		282 (98%)		0.173	X^2^
(+)	1 (0.5%)		6 (2%)			

Bold italics indicate statistical significance (*p* < 0.05)

*uURS* urgent ureteroscopy, *eURS* elective ureteroscopy, *X*^*2*^ Ki-Kare test, *m* Mann–Whitney *u* test

Although there was a statistically significant difference between the groups in terms of urine culture and urine test, no statistically significant difference in urine culture and urinary nitrite positivity (*p* > 0.05) (Table 4). Urine cultures were studied from two patients who needed postoperative intensive care unit (ICU) follow-up, and only one showed growth (per-op urine culture), while urine analysis was performed in both patients and urinary nitrite was not positive. Likewise, urinalysis was performed on all patients with high fever and it was observed that nitrite positivity was not detected in the urine. Although this situation does not show a statistically significant difference, it suggests that urine culture is more successful in predicting sepsis, especially in patients with suspected sepsis.

According to the Clavian Dindo classification, 7 patients had grade I, 18 patients grade III and only 2 patients had grade IV complications (Tables 3 and 5).

**Table 5:** Comparison of stone characteristics, operative variables and complications

	uURS		eURS		*p*	
	Mean ± sd/*n* (%)	Median	Mean ± sd/*n* (%)	Median		
Stone Size (mm)	7.46 ± 2.20	7.00	8.44 ± 2.79	8.00	***0.000***	m
Stone location						
Proximal	22 (10.0%)		72 (20.4%)		***0.002***	X^2^
Middle	37 (16.9%)		75 (21.2%)		0.243	X^2^
Distal	128 (58.4%)		171 (48.4%)		***0.024***	X^2^
Ureterovesical junction	32 (14.6%)		35 (9.9%)		0.178	X^2^
Side of stone						
Right	107 (48.9%)		161 (45.6%)		0.449	X^2^
Left	112 (51.1%)		192 (54.4%)			
Stenting						
No	57 (26.0%)		75 (21.2%)		0.187	X^2^
Yes	162 (74.0%)		278 (78.8%)			
Stone free						
Yes	202 (92.2%)		300 (85.0%)		***0.010***	X^2^
No	17 (7.8%)		53 (15.0%)			
Failure of access						
No	216 (98.6%)		333 (94.3%)		***0.011***	X^2^
Yes	3 (1.4%)		20 (5.7%)			
Reason for failed access						
Tight ureter	3 (1.8%)		11 (3%)		0.254	X^2^
Mucosal oedema	0 (0.0%)		3 (0.8%)		0.290	X^2^
Haematuria	0 (0.0%)		1 (0.3%)		1.000	X^2^
Impacted stone	0 (0.0%)		5 (1.4%)		0.162	X^2^
Complications						
Yes	8 (3.7%)		19 (5.4%)		0.343	X^2^
No	211 (96.3%)		334 (94.6%)			
Hematuria >24 h	0 (0.0%)		3 (0.8%)		0.290	X^2^
Fever	1 (0.5%)		3 (0.8%)		1.000	X^2^
Stone Migration	3 (1.4%)		11 (3.1%)		0.189	X^2^
Intensive Care Unit	2 (0.9%)		0 (0.0%)		0.146	X^2^
Renal colic	2 (0.9%)		2 (0.6%)		0.639	X^2^
Operative time (min)	37.2 ± 9.0	35.0	41.2 ± 9.8	40.0	***0.000***	m
Length of hospital stay	1.33 ± 0.65	1.00	1.61 ± 0.62	2.00	***0.000***	m

Bold italics indicate statistical significance (*p* < 0.05)

*uURS* urgent ureteroscopy, *eURS* elective ureteroscopy, *X*^*2*^ Ki-Kare test, *m* Mann–Whitney *u* test

## Discussion

The causes of stone formation are multifactorial and develop depending on genetic, ethnic, geographical conditions and nutritional habits [8]. Being in a hot climate zone and high quality of life causes an increase in stone prevalence. The overall prevalence varies between 1 and 20% [8–11]. Obesity, which is one of the most important diseases that has been increasing in recent years and threatening health, and in parallel with the sedentary lifestyle and fast-food culture that has increased in this way, stone disease is increasing day by day [5, 12, 13]. Depending on this situation, there is an increase in the rate of admission to the emergency department or urology department due to renal colic [14, 15].

The first thing to do in patients with renal colic is to relieve the pain [16]. For this purpose, while non-steroidal anti-inflammatory drugs (diclofenac, ibuprofen, ketorolac, etc.) are used most often, paracetamol, antispasmodic and opioid type drugs are also used [4, 5]. After analgesia is planned, definitive treatment planning is made according to the patient’s clinical, imaging and laboratory evaluations. Treatment planning varies depending on the location, size, number and structure of the stone. In addition to stone-related parameters, the choice is made considering the accompanying comorbidity and patient preference.

In case of colic pain resistant to analgesics, anuria and sepsis a percutaneous nephrostomy or ureteral JJ stent is placed and a final treatment planning is made after stabilization. Although there are some studies showing that either double jj stent or percutaneous nephrostomy is superior to the other, their overall effectiveness and safety have been shown to be similar. While percutaneous nephrostomy has the advantage of being performed faster with local anesthesia without the need for general anesthesia, it can be said to be disadvantageous when evaluated in terms of patient comfort. On the other hand, the need for anesthesia for the double jj stent and the inability to place a guide wire or double jj stent in cases of tight and edematous ureter and impacted stones due to the stone not being able to be passed are among its disadvantages. However, it is relatively more comfortable than percutaneous nephrostomy and provides passive dilatation for URS to be performed later. It can be said as an advantage [4, 13, 16–18]. If the patient is clinically stable and there is no suspicion of sepsis, the classical treatment of renal colic is MET, ESWL, URS and laparoscopic/open surgery according to stone-related factors [1, 19]. Except for laparoscopic/open surgeries, which are performed less frequently, limited to larger and more complicated stones, the other three treatments are considered in the first place. Although it is more invasive, rapid pain relief and stone clearance make URS advantageous [5, 11, 12].

Ureteroscopy can be performed as eURS or uURS. Elective URS is performed after the necessary preparations are made in patients who were initially treated with MET and ESWL but failed [20]. In this process, some patients often experience repeated hospital admissions, mostly due to pain. This reality is not a desirable situation for both patients and physicians. Therefore, if the patient is stable in renal colic due to ureteral stones, uURS may be recommended as the primary treatment [18, 21].

The stone-free rate in elective URS is over 90% [11, 16, 20]. The advantages of miniaturization and flexibility, especially in instruments, and improvements in other instruments such as lithotripters and forceps further increase the success rate of URS, which is high. Despite the positive developments in all these technologies, the success rate for proximal ureteral stones is still slightly lower. Due to the increase in diameter of ureterorenoscopes from distal to proximal, snagging at the ureteral orifice, especially the kink in the mid-proximal ureter, makes it difficult to place the ureterorenoscope. In addition, although it is less common with laser lithotriptors, retropulsion of the stone is more likely, especially if pneumatic lithotriptors are used. An anti-retropulsion device can be used to prevent the stone from being pushed back (it may restrict the movement of the ureterorenoscope), or this problem can be prevented by adjusting the energy and frequency of the laser. On the other hand, if you have a flexible ureterorenoscope, the stone can be fragmented by providing easy access even if it is push-back [7, 19, 22, 23]. In our study, a success rate of 92.2% was obtained in uURS, similar to eURS, and this was confirmed (Table 5). With uURS, the patient’s pain can be relieved quickly, and recurrent admissions, increased analgesic use and secondary morbidities, prolonged hospital stays (shorter in uURS in our study) and related costs and extended surgery appointment days are prevented [4, 9, 22, 24]. In addition, it does not require additional treatment compared to MET and SWL treatment, which is another advantage. Thus, further labor loss is prevented and cost-effective treatment is provided by reducing costs [1, 24, 25]. Another important point is that the renal damage due to obstruction reaches 30% within 15 days, and only 70% recovers after the obstruction is removed. For this reason, it is crucial to ensure rapid stone clearance [1]. On the other hand, the rapid return to work and social life is another very important advantage of uURS[15].

At this point, it is a dilemma to have over-treatment for stones that may pass spontaneously or after MET, by applying a procedure that requires anesthesia with a general complication rate of 9–25% [11, 22]. Therefore, patient selection should be made very carefully and treatment options should be presented to patients in detail, and uURS should be applied as a physician–patient joint decision in the selected patient population [22].

Studies conducted in terms of complications have shown that there is no difference between uURS and eURS results [9, 16]. Considering the complications of ureterorenoscopy, there are minor complications such as fever, stent-related discomfort, and hematuria, but there may also be serious major complications such as ureteral avulsion <1%. Similarly, in our study, both procedures were found to be similar in terms of complications (Table 5).

Fever is the most important parameter for postoperative sepsis and should be followed very carefully and closely, and if necessary, early ICU follow-up should be performed by expanding the antibiotic spectrum in a timely manner [26]. In two of our patients who underwent uURS, after the development of sepsis, the treatment regimen was quickly reorganized and they were followed up with ICU, and their treatment was successfully provided. Urine culture is the most important parameter in predicting urosepsis, which causes mortality up to 26% if not treated appropriately. As the result of the gold standard urine culture will take 24 h at the earliest, which patient can go to sepsis will depend on the clinician’s evaluation in the light of clinical, laboratory and imaging studies. In patients with high acute phase reactants, fever, kidney failure, and pyuria and nitrite positivity in urine analysis, urine culture should be taken and broad-spectrum empirical antibiotic treatment should be started, and antibiotic changes should be made if necessary, according to the urine culture results [27]. Definitive treatment should be left after clinical stabilization. As stated in the American Urological Association Endourological Society Guideline, in our current study, urinalysis was found to be sufficient in the patient group who were clinically stable and had no suspicion of infection or sepsis [28].

Our present study is not devoid of limitations. First of all, the main limitation of our study is that it is not in a prospective and randomized controlled format. Lack of stone density and analysis, quality of life score, and lack of long-term results such as ureteral stricture and ectasia are other important limitations. Other limitations include making cost-effectiveness comparisons based on other studies and using our own subjective predictions, and not comparing one-to-one with rational data.

## Conclusion

In clinically stable patients without suspected sepsis, elective treatment planning can be made, or uURS can be performed effectively and safely as a definitive single-stage primary treatment, especially in distal ureteral stones. In this way, recurrent hospital admissions, examination and medication are prevented by performing the primary treatment of the patient. The patient’s admission to the hospital with more symptoms or appointments, thus preventing the burden of urolithiasis on both the individual and the health system. It would be beneficial to support this conclusion with large series, prospective, randomized controlled studies.

## Data Availability

All data supporting the findings of this study are available within the paper and its Supplementary Information.

## References

[1] Guercio S, Ambu A, Mangione F, Mari M, Vacca F, Bellina M (2011) Randomized prospective trial comparing immediate versus delayed ureteroscopy for patients with ureteral calculi and normal renal function who present to the emergency department. J Endourol 25:1137–1141. 10.1089/end.2010.0554 21682597 10.1089/end.2010.0554

[2] Picozzi SCM, Ricci C, Gaeta M, Casellato S, Stubinski R, Ratti D, Bozzini G, Carmignani L (2012) Urgent shock wave lithotripsy as first-line treatment for ureteral stones: a meta-analysis of 570 patients. Urol Res 40:725–731. 10.1007/s00240-012-0484-0 22699356 10.1007/s00240-012-0484-0

[3] Teichman JMH (2004) Clinical practice. Acute renal colic from ureteral calculus. N Engl J Med 350:684–693. 10.1056/NEJMcp030813 14960744 10.1056/NEJMcp030813

[4] Jung H, Osther PJS (2015) Acute management of stones: when to treat or not to treat? World J Urol 33:203–211. 10.1007/s00345-014-1353-y 24985553 10.1007/s00345-014-1353-y

[5] Makanjuola JK, Rintoul-Hoad S, Bultitude M (2016) Evolving guidance on ureteric calculi management in the acute setting. Curr Urol Rep 17:24. 10.1007/s11934-016-0574-6 26874536 10.1007/s11934-016-0574-6

[6] Zhang Z, Wang X, Chen D, Peng N, Chen J, Wang Q, Yang M, Zhang Y (2020) Minimally invasive management of acute ureteral obstruction and severe infection caused by upper urinary tract calculi. J Xray Sci Technol 28:125–135. 10.3233/XST-190576 31796723 10.3233/XST-190576

[7] Kandasami SV, Mamoulakis C, El-Nahas AR, Averch T, Tuncay OL, Rawandale-Patil A, Cormio L, de la Rosette JJ, CROES URS Global Study Group (2014) Impact of case volume on outcomes of ureteroscopy for ureteral stones: the clinical research office of the endourological society ureteroscopy global study. Eur Urol 66:1046–1051. 10.1016/j.eururo.2014.06.054 10.1016/j.eururo.2014.06.054 25027366

[8] European Association of Urology (2023) Urolithiasis. https://uroweb.org/guidelines/urolithiasis. Accessed 2 Aug 2023

[9] Mckay A, Somani BK, Pietropaolo A, Geraghty R, Whitehurst L, Kyriakides R, Aboumarzouk OM (2021) Comparison of primary and delayed ureteroscopy for ureteric stones: a prospective non-randomized comparative study. Urol Int 105:90–94. 10.1159/000510213 32894854 10.1159/000510213

[10] Ordonez M, Hwang EC, Borofsky M, Bakker CJ, Gandhi S, Dahm P (2019) Ureteral stent versus no ureteral stent for ureteroscopy in the management of renal and ureteral calculi. Cochrane Database Syst Rev 2019:CD012703. 10.1002/14651858.CD012703.pub2 10.1002/14651858.CD012703.pub2 PMC6365118 30726554

[11] Picozzi SCM, Ricci C, Gaeta M, Casellato S, Stubinski R, Bozzini G, Pace G, Macchi A, Carmignani L (2012) Urgent ureteroscopy as first-line treatment for ureteral stones: a meta-analysis of 681 patients. Urol Res 40:581–586. 10.1007/s00240-012-0469-z 22367457 10.1007/s00240-012-0469-z

[12] Wani M, Burki J, Melhem M, Gilani S, Ghumman F, Masood S (2021) Is primary ureteroscopy an alternative to emergency stenting in terms of quality and cost? Cent European J Urol 74:446–450. 10.5173/ceju.2021.0029.R1 34729235 10.5173/ceju.2021.0029.R1 PMC8552929

[13] Pietropaolo A, Hendry J, Kyriakides R, Geraghty R, Jones P, Aboumarzouk O, Somani BK (2020) Outcomes of elective ureteroscopy for ureteric stones in patients with prior urosepsis and emergency drainage: prospective study over 5 yr from a tertiary endourology centre. Eur Urol Focus 6:151–156. 10.1016/j.euf.2018.09.001 30219711 10.1016/j.euf.2018.09.001

[14] Al-Terki A, Alkabbani M, Alenezi TA, Al-Shaiji TF, Al-Mousawi S, El-Nahas AR (2020) Emergency vs elective ureteroscopy for a single ureteric stone. Arab J Urol 19:137–140. 10.1080/2090598X.2020.1813004 34104487 10.1080/2090598X.2020.1813004 PMC8158266

[15] Dauw CA, Kaufman SR, Hollenbeck BK, Roberts WW, Faerber GJ, Wolf JS, Hollingsworth JM (2014) Expulsive therapy versus early endoscopic stone removal in patients with acute renal colic: a comparison of indirect costs. J Urol 191:673–677. 10.1016/j.juro.2013.09.028 24060643 10.1016/j.juro.2013.09.028

[16] Arcaniolo D, De Sio M, Rassweiler J, Nicholas J, Lima E, Carrieri G, Liatsikos E, Mirone V, Monga M, Autorino R (2017) Emergent versus delayed lithotripsy for obstructing ureteral stones: a cumulative analysis of comparative studies. Urolithiasis 45:563–572. 10.1007/s00240-017-0960-7 28233025 10.1007/s00240-017-0960-7

[17] Joshi HB, Obadeyi OO, Rao PN (1999) A comparative analysis of nephrostomy, JJ stent and urgent in situ extracorporeal shock wave lithotripsy for obstructing ureteric stones. BJU Int 84:264–269. 10.1046/j.1464-410x.1999.00174.x 10468719 10.1046/j.1464-410x.1999.00174.x

[18] Pietropaolo A, Seoane LM, Abadia AA-S, Geraghty R, Kallidonis P, Tailly T, Modi S, Tzelves L, Sarica K, Gozen A, Emiliani E, Sener E, Rai BP, Hameed ZBM, Liatsikos E, Rivas JG, Skolarikos A, Somani BK (2022) Emergency upper urinary tract decompression: double-J stent or nephrostomy? A European YAU/ESUT/EULIS/BSIR survey among urologists and radiologists. World J Urol 40:1629–1636. 10.1007/s00345-022-03979-4 35286423 10.1007/s00345-022-03979-4 PMC8918906

[19] Perez Castro E, Osther PJS, Jinga V, Razvi H, Stravodimos KG, Parikh K, Kural AR, de la Rosette JJ, CROES Ureteroscopy Global Study Group (2014) Differences in ureteroscopic stone treatment and outcomes for distal, mid-, proximal, or multiple ureteral locations: the Clinical Research Office of the Endourological Society ureteroscopy global study. Eur Urol 66:102–109. 10.1016/j.eururo.2014.01.011 10.1016/j.eururo.2014.01.011 24507782

[20] Nestler S, Grüne B, Schilchegger L, Neisius A, Jones J (2021) Evaluation of stone free rates in early versus delayed primary ureteroscopy: time does matter. World J Urol 39:909–914. 10.1007/s00345-020-03235-7 32488357 10.1007/s00345-020-03235-7

[21] Bakr M, Abdelhalim KM (2020) Safety and efficacy of emergency ureteroscopy with intracorporeal lithotripsy in patients presented with urinary tract infection with mild sepsis. J Endourol 34:262–266. 10.1089/end.2019.0550 31989843 10.1089/end.2019.0550

[22] Bangash M, Nazim SM, Khan N, Ghani O, Naeem S (2022) Comparison of emergency and elective intervention with semi-rigid ureteroscopic lithotripsy for patients with ureteral calculi. J Ayub Med Coll Abbottabad 34:67–72. 10.55519/JAMC-01-9612 10.55519/JAMC-01-9612 35466630

[23] Hiller SC, Ghani KR (2020) Frontiers of stone management. Curr Opin Urol 30:17–23. 10.1097/MOU.0000000000000698 31725002 10.1097/MOU.0000000000000698

[24] Somani BK, Bres-Niewada E (2021) Primary ureteroscopy versus emergency stenting and delayed ureteroscopy: is there a winner? Cent European J Urol 74:451–452. 10.5173/ceju.2021.Ed1 34729236 10.5173/ceju.2021.Ed1 PMC8552927

[25] Elderwy AA, Gadelmoula M, Elgammal MA, Hameed DA, Behnsawy HM, Osman MM, Kurkar A (2018) Primary versus deferred ureteroscopy for management of calculus anuria: a prospective randomized study. Cent European J Urol 71:462–466. 10.5173/ceju.2018.1768 30680242 10.5173/ceju.2018.1768 PMC6338810

[26] Risk factors for urosepsis after ureteroscopy for stone disease: a systematic review with meta-analysis. https://www.liebertpub.com/doi/epdf/10.1089/end.2020.1133. Accessed 22 Aug 2022 10.1089/end.2020.1133 33544019

[27] Grossmann NC, Schuettfort VM, Betschart J, Becker AS, Hermanns T, Keller EX, Fankhauser CD, Kranzbühler B (2022) Risk factors for concomitant positive midstream urine culture in patients presenting with symptomatic ureterolithiasis. Urolithiasis 50:293–302. 10.1007/s00240-022-01323-4 35441879 10.1007/s00240-022-01323-4 PMC9110449

[28] Assimos D, Krambeck A, Miller NL, Monga M, Murad MH, Nelson CP, Pace KT, Pais VM, Pearle MS, Preminger GM, Razvi H, Shah O, Matlaga BR (2016) Surgical management of stones: american urological association/endourological society guideline, PART I. J Urol 196:1153–1160. 10.1016/j.juro.2016.05.090 27238616 10.1016/j.juro.2016.05.090

